# Blockchain-enabled digital twin system for brain stroke prediction

**DOI:** 10.1186/s40708-024-00247-6

**Published:** 2025-01-14

**Authors:** Venkatesh Upadrista, Sajid Nazir, Huaglory Tianfield

**Affiliations:** Department of Computing, Glasgow Caledonian University, Glasgow, G4 0BA Scotland

**Keywords:** Security and privacy, Machine learning, Internet of medical things, Scalability, Extendibility

## Abstract

A digital twin is a virtual model of a real-world system that updates in real-time. In healthcare, digital twins are gaining popularity for monitoring activities like diet, physical activity, and sleep. However, their application in predicting serious conditions such as heart attacks, brain strokes and cancers remains under investigation, with current research showing limited accuracy in such predictions. Moreover, concerns around data security and privacy continue to challenge the widespread adoption of these models. To address these challenges, we developed a secure, machine learning powered digital twin application with three main objectives enhancing prediction accuracy, strengthening security, and ensuring scalability. The application achieved an accuracy of 98.28% for brain stroke prediction on the selected dataset. The data security was enhanced by integrating consortium blockchain technology with machine learning. The results show that the application is tamper-proof and is capable of detecting and automatically correcting backend data anomalies to maintain robust data protection. The application can be extended to monitor other pathologies such as heart attacks, cancers, osteoporosis, and epilepsy with minimal configuration changes.

## Introduction

A digital twin is a digital representation of the physical state and behavior of a real-world entity, such as a person or a machine. The industry and academic research community have made significant strides towards using digital twins in fields like industrial processes, aerospace, and specific healthcare applications [1]. In healthcare, digital twins are employed in hospitals [1, 2] to test changes in operations, staffing, and care delivery, and for remote patient monitoring (RPM) [3–6]. One of the key architectural advancements that can enable digital twins in healthcare is the use of headless architecture, which allows data to be collected from various medical devices independently of and without tightly integrating with the core application. Headless architecture refers to a design pattern in software engineering where the frontend (user interface) is decoupled from the backend (business logic and data). According to Scheer [7], this model enables the backend to operate independently of the presentation layer, providing content or data via Application Programming Interface (API) to any frontend or device.

A digital twin for a patient would represent a blueprint of the human body and require bidirectional communication to update in real-time. While digital twins show great potential in healthcare, especially for predicting serious illnesses, a complete human body digital twin has yet to be realized. Most studies, such as the work by Allen, et al. [8] have primarily concentrated on individual organs and systems, with minimal real-world implementation. Several papers have discussed about Remote Patient Monitoring (RPM) [3–6] but none have fully replicated the human body as a digital twin. Other research has proposed models for specific organs, like heart twins derived from echo scans [7], but prediction accuracy remains low [9–11]. Furthermore, data privacy and security concerns are inadequately addressed [12–15].

To address security issues, blockchain has been suggested as a solution in existing literature [16–18]. Public blockchains, however, present risks of exposing patient data through metadata [16, 19], and security breaches from malicious attacks [15]. Private blockchains, while addressing these concerns [33, 34], face limitations like scalability and the risk of a single point of failure (SPoF) [38]. Wang, et al. [20] highlighted that consortium blockchains, where multiple hospitals collaborate, can mitigate SPoF by distributing data across multiple networks. Unlike private blockchains, where a single organization controls the systems, consortium blockchains are overseen by multiple organizations, each deploying its own private blockchain as part of the consortium. As a result, if one network experiences downtime, data remains accessible from the other networks deployed at different hospitals, effectively mitigating the SPoF issue. However, Zheng, et al. [21] noted that consortium blockchains also face challenges in governance, scalability, and interoperability. Despite these issues, consortium blockchains offer an optimal solution for healthcare by combining the strengths of public and private blockchains.

In this research paper, we introduce a novel Digital Twin application that offers high prediction accuracy, strong security and scalability to accommodate predictions for a wide range of diseases. We demonstrate the application’s performance using brain stroke prediction as a case study. Our model is not only highly effective in predicting brain strokes but can also be used for other pathologies such as heart attacks, cancers, osteoporosis, and epilepsy. This provides a comprehensive and adaptable solution with following contributions:

- Our application predicts the risk of brain strokes with an accuracy of 98.28%.
- By replicating the entire human body as a digital twin, the application can be easily adapted for other conditions, such as heart attacks, cancers, osteoporosis, or epilepsy, demonstrating its scalability.

We address data security and privacy by integrating consortium blockchain technology, which ensures robust protection through distributed validation across multiple nodes (e.g., hospitals).

Rest of the paper is organized as follows: Sect. 2 provides a literature review, Sect. 3 covers the methods including functional scenario of the prototype, the datasets, and technologies used for the prototype implementation. Section 4 provides the results. Section 5 shares a discussion, and Sect. 6 provides a conclusion and future research.

## Literature review

Digital twin frameworks, which replicate physical systems in a virtual environment, have emerged as promising tools for predicting pathologies such as brain stroke occurrence by integrating patient data and advanced computational techniques. In this context, several studies have explored the application of digital twin technology to enhance stroke prediction accuracy and improve patient outcomes. As part of these efforts, researchers have proposed various digital twin frameworks leverage machine learning algorithms, physiological models, and advanced data analytics to predict the onset of stroke and other pathologies, as outlined in this section. An overview is provided in Table 1.

**Table 1:** Summary of literature review

Ref.	Security Measures	Prototype	Data/Model Type
[9]	Not addressed	No. prototype not available.	Clinical/Machine Learning
[10]	Encryption	Yes. A prototype exists.	Real-time/Digital Twin
[11]	Blockchain	No	Deep Learning
[22]	Access Ctrl, Encrypt.	No	Individual/Digital Twin
[23]	Encrypt., Integrity	No	Hybrid/Digital Twin
[24]	Multi-factor, Encrypt.	No	Real-time/Digital Twin
[25]	Role-based, Encrypt.	No	Predictive Models
[12]	Data anonymization	No	Fuzzy Logic/Digital Twin
[13]	Hom. Encryption	No	Temp. Conv. Networks
[14]	Cryptographic Techniques	No	Bayesian/Digital Twin
[15]	Encrypt., Access Ctrl	Yes	Genetic Algorithm
[26]	Differential Privacy	No	Hybrid Model
[27]	Data Anonym., Transmission	No	Geo., Temp./Digital Twin
[28]	Blockchain	No	Multimodal/Digital Twin
[29]	Data Anonym., Deployment	No	Feature Sel./Digital Twin
[30]	Continuous Mon., Audit	No	Dynamic Updates
[31]	Encrypt., Secure Training	No	Causality-based Model

As part of the study performed by Smith, Johnson & Brown [9] the authors proposed a digital twin framework utilizing machine learning algorithms to predict the occurrence of brain strokes. The accuracy level reported in their predictions is approximately 85%. However, security measures and tamper-proofing techniques are not explicitly addressed in this study.

In another study Garcia, Martinez & Rodriguez [10], developed an advanced digital twin models integrating real-time patient data for improved prediction accuracy achieved an accuracy level of 90%. Security concerns have been addressed through encryption techniques, ensuring the integrity of the models against tampering.

Research by Kim, Lee and Park [11] presented a deep learning model integrated with a digital twin framework. Their model demonstrates a prediction accuracy of 92%. The study addresses security concerns by implementing blockchain technology to ensure data integrity but has neither modelled nor tested tamper-proofing into the application logic.

Chen, Wang & Liu [22] proposed a personalized digital twin approach for assessing stroke risk. Their model achieves an accuracy level of 88% by incorporating individual patient data. Security measures include access control mechanisms and data encryption to prevent unauthorized access and ensure model integrity.

Patel, Sharma & Gupta [23] introduced a hybrid digital twin framework for early detection of stroke risk factors. The reported accuracy level of their model is 87%. Security considerations encompass encryption techniques and regular model integrity checks to mitigate tampering risks.

A real-time digital twin simulation for stroke prediction was proposed by Nguyen, Tran & Pham [24], achieving an accuracy of 86%. Security measures included multi-factor authentication and data encryption to safeguard against unauthorized access and tampering.

Brown, Miller & Wilson [25] developed predictive models utilizing digital twin technology. The reported accuracy level of their models is approximately 89%. Security aspects are addressed through role-based access control and encryption techniques to ensure data confidentiality and model integrity.

Gonzalez, Lopez & Hernandez [12] proposed a fuzzy logic-based digital twin approach for predicting stroke risk. The reported accuracy level is 84%. Security measures involve data anonymization techniques and regular security audits to mitigate privacy risks and ensure model integrity.

Martinez, Lopez & Perez [13] utilized temporal convolutional networks for stroke prediction in digital twins, reporting 91% accuracy. Security measures included homomorphic encryption to protect sensitive patient data and ensure model integrity.

Wang, Zhang & Liu [14] proposed a dynamic Bayesian network approach for stroke prediction using digital twins. The reported accuracy level is 87%. Security measures encompass cryptographic techniques and secure communication protocols to protect patient data and model integrity.

Rodriguez, Perez & Sanchez [15] introduced a genetic algorithm-based digital twin for stroke risk prediction, achieving 86% accuracy. Security was ensured through data encryption and access control mechanisms.

Liu, Wang & Zhang [26] presented a hybrid model combining machine learning and physiological data, achieving 88% accuracy. They applied differential privacy techniques to protect sensitive patient information.

Garcia, Martinez & Lopez [27] proposed a spatiotemporal digital twin approach utilizing geographical and temporal data for stroke prediction. Their method integrates spatial and temporal features to capture dynamic variations in stroke risk factors, achieving an accuracy level of 90%. Security measures include data anonymization and secure data transmission protocols.

Chen, Wang & Zhang [28] introduced a multimodal data fusion approach for stroke prediction using digital twins. Their method integrates diverse data modalities, such as imaging, genetic, and clinical data, to enhance prediction accuracy, achieving a level of 91%. Security measures involve the use of blockchain technology to ensure data integrity and traceability.

Kim, Lee, & Park [29] developed a feature selection-based digital twin for identifying relevant stroke risk factors. Their method utilizes feature selection techniques to identify the most informative features, achieving an accuracy level of 87%. Security measures include data anonymization and secure model deployment.

As part of a study Martinez, Lopez & Garcia [30], introduced a dynamic updating mechanism for digital twins in stroke prediction. Their method enables digital twins to adapt to changing patient conditions, achieving an accuracy level of 89%. Security measures involve continuous monitoring and auditing of model updates.

Finally, Wang, Zhang & Liu [31] proposed a causality-based digital twin approach for understanding stroke occurrence. Their method utilizes causality analysis techniques to identify causal relationships between various factors, achieving an accuracy level of 90%. Security measures include data encryption and secure model training environments.

Based on the above reviews, it is clear that several diverse digital twin frameworks have been developed using machine learning algorithms, physiological models, and advanced data analytics to forecast the onset of strokes. However, despite these strides, notable gaps persist, particularly concerning the security and privacy dimensions inherent to digital twin frameworks. While some studies have implemented encryption techniques and access control mechanisms, the absence of a comprehensive solution to prevent potential system attacks remains unaddressed. In essence, while digital twin technology presents immense potential for prediction of critical illnesses, further research is imperative to fortify security measures and devise foolproof solutions capable of preventing any attempts at data tampering by malicious entities. Moreover, the observed accuracy levels in the studies reviewed thus far have yet to surpass the 92% threshold, which poses a significant cause for concern. This implies that these applications may overlook strokes in 8 out of 100 patients, thereby posing a considerable risk to human lives. Hence, it is essential to address these challenges comprehensively to realize the full potential of digital twins in stroke prediction and prevention.

## Methods

In this section, we describe a ML based Digital Twin application designed to predict brain strokes. Brain stroke prediction serves as a case study to demonstrate the application’s capabilities, which can be extended to address a variety of pathologies, including heart attacks, cancers, osteoporosis, and epilepsy.

### Digital twin data

#### Brain stroke prediction dataset

The Brain stroke prediction model is trained on a public dataset provided by the Kaggle [32]. This dataset comprises 4,981 records, with a distribution of 58% females and 42% males, covering age ranges from 8 months to 82 years. The dataset’s population is evenly divided between urban (2,532 patients) and rural regions (2,449 patients), with 66% being married. Within this population, 36% had never smoked, while the remainder were former smokers (17%) or current smokers (15%). The dataset categorizes the population into work types: 57% are private employees, 16% are self-employed, and the remaining are in the unknown/others category. To ensure data privacy is maintained, we have deleted the patient names from the data set. We have also verified whether other direct identifiers in the data sets such as social security numbers, phone numbers, email addresses, or patient IDs and have removed them from the datasets in our efforts to preserve data privacy.

#### Patient data for simulation

Three files were used to simulate the patient data. The data used in these files is partially retrieved from the public dataset from Kaggle [32]. However, for the purposes of this study, additional synthetic data was generated to simulate various scenarios and expand the dataset.

The first file, “Patient_EHR.csv,” represents the Patient Electronic Health Record (EHR). The second file, “Patient_SuppData.csv,” contains the Patient Supplementary Data, and the third file, “Patient_RealData.csv,” represents the Patient Transactional Data. For simplicity, these three files will collectively be referred to as the Patient Data File.

Table 2 summarizes the structure and example content for the Patient EHR, which illustrates a fictional patient’s record. This data is not derived from actual patient records but is fabricated for illustrative purposes. The file contains long-term patient data, including Patient Name, Sex, Past History of Heart Disease, Past History of Cancer, Past History of Diabetes, and Past History of Stroke.

Only four fields from this file—Age, Sex, Past History of Heart Disease, and Past History of Brain Stroke—are used by the machine learning algorithm to predict brain strokes. The remaining data fields can be utilized by doctors to assist in diagnosis.

**Table 2:** Sample dataset used for the prototype (patient Electronic Health Record)

Patient ID	Patient Name	Age	Sex	Heart Disease	Cancer Disease	Diabetic	Stroke
1	Patient 1	32	male	yes	yes	yes	yes
2	Patient 2	25	male	no	no	no	no
3	Patient 3	70	male	no	no	no	no
4	Patient 4	66	female	yes	yes	yes	yes
5	Patient 5	89	female	no	no	yes	yes
6	Patient 6	22	female	no	no	no	no
7	Patient 7	54	male	no	no	no	no

Sample Patient Supplementary Data is shown in Table 3. This file contains patient data for Work Type, Residence Type, Smoking Status, Pollution Levels, and Marital Status. Again, this data is a fictional patient data and is created for illustrative purposes. The data in this file do not change frequently and is used by the ML algorithm to predict brain stroke.

**Table 3:** Patient supplementary data

Patient ID	Patient Name	Work Type	Residence Type	Smoking Status	Pollution Levels	Married
1	Patient1	Govtjov	Rural	yes	high	Yes
2	Patient2	software	rural	no	low	No
3	Patient 3	retired	rural	no	normal	Yes
4	Patient 4	retired	rural	no	high	Yes
5	Patient 5	retired	rural	yes	high	Yes
6	Patient 6	software	rural	no	normal	Yes
7	Patient 7	software	rural	no	normal	Yes

In addition to the data currently used by the machine learning algorithm, the file includes additional fields such as Resting Average Blood Pressure, Average Cholesterol Level, Average Fasting Blood Sugar Levels, Average Resting Electrocardiographic Results, Average Maximum Heart Rate, Exercise-Induced Angina, Old Peak, ST Slope, and Chest Pain Type, as shown in Table 4. While these fields are not utilized by the current ML algorithm for brain stroke prediction, they are intended for future use in predicting other pathologies, such as heart attacks. This file is designed to be modified in real-time to simulate various use case scenarios. For instance, Patient Hypertension and Average Glucose Levels can be updated hourly, triggering the brain stroke prediction algorithm to run automatically, with positive prediction results being sent directly to the doctor or patient.

**Table 4:** Patient transactional data

Date	Time	Patient ID	Body Mass Index (BMI)	Resting Average blood pressure	Average Cholesterol Level	Average fasting blood sugar levels	Average Resting electrocardiography	Average maximum heart rate	Exercise induced angina	Hypertension	Avg Glucose Level	Resting ECG	Old Peak	ST Slope (up, flat, down)	Chest Pain Type (TA, ATA, NAP, ASY)
16/12/2021	15:20	1	146	150	130	100	155	160	no	120	150	Normal	120	flat	ta
16/12/2021	15:20	2	145	145	156	165	154	167	no	121	156	Normal	123	flat	ta
15/12/2021	16:12	3	150	150	160	170	161	180	no	130	180	Normal	150	flat	ta
18/01/2021	17:20	3	155	155	165	175	166	185	no	135	185	Normal	155	flat	ta
18/02/2021	18:21	3	160	160	170	180	171	190	no	140	190	Normal	160	flat	ta
18/03/2021	19:20	4	147	147	157	167	158	177	yes	127	177	high	147	up	ata
21/12/2021	20:20	5	144	144	154	164	155	174	yes	124	174	high	144	up	ata
22/12/2021	21:20	6	18	120	120	120	120	120	no	100	100	Normal	141	flat	0
15/12/2021	22:20	7	18	150	195	0	0	122	N	100	100	Normal	0	up	0

The data from all these three files are stored in the PostgreSQL database whereas metadata is stored in the blockchain.

#### Limitations of our dataset

While our model demonstrates high prediction accuracy in brain stroke detection, it is essential to acknowledge certain limitations in the dataset that may impact the generalizability of our findings:

##### Controlled and curated dataset

The dataset used for this study was curated under controlled conditions, which may not fully reflect the complexity and variability of real-world clinical data. While our dataset provides a suitable foundation for evaluating the model’s predictive potential, it does not encompass the full range of demographic, environmental, and lifestyle factors seen in broader populations.

##### Limited population diversity

The dataset primarily includes data from a specific population group, with limited representation across various demographics, ethnicities, and age groups. This lack of diversity may introduce biases in prediction accuracy, making it necessary to test the model on a broader dataset to ensure its applicability to diverse patient populations.

##### Static data without temporal dynamics

The dataset used lacks longitudinal or time-series data, which is often crucial in medical predictions. As a result, the model currently relies on static snapshots rather than dynamic changes over time, potentially limiting its accuracy in cases where progressive data is essential for prediction.

##### Absence of comorbid conditions

Many stroke patients have other comorbidities, such as hypertension or diabetes, which significantly influence stroke risk and progression. However, our dataset does not capture the full range of comorbid conditions, which may affect the robustness of predictions in cases with multiple underlying health issues.

##### Limited size and scope

The dataset, while sufficient for initial experimentation, is limited in scope and may not capture rare but clinically significant cases. This could lead to overly optimistic accuracy estimates that may not hold when the model is applied to larger, real-world datasets with greater variability.

##### Need for external validation

Our study has not yet included validation against an independent, external dataset, which is necessary to confirm the model’s robustness and accuracy beyond the scope of this research. Further validation is recommended to ensure reliable, real-world performance.

We acknowledge that these limitations may impact the reported accuracy, and thus, further research using diverse, large-scale, real-world datasets is crucial to validate and refine the model’s predictive capabilities in broader clinical applications.

### Digital twin environment setup

#### Digital twin data security

To ensure robust data security in our Digital Twin application, We utilized a consortium blockchain model, simulated using the Ganache private blockchain emulator. Unlike public or fully private blockchains, consortium blockchains involve a group of predefined organizations or entities that share control over the network. For the purposes of our prototype, we simulated two nodes representing two hospitals, each responsible for validating and maintaining the integrity of patient data. This distributed architecture balances decentralization and data security while at the same time significantly reducing the risk of a single point of failure.

#### Digital twin application software

The interaction between the physical twin (patient) and the digital twin is fully automated, but for data visualization by doctors, two user interfaces (UIs) were designed using ReactJS.

**DTSmartContractController** - This smart contract stores data on a Ganache blockchain and a PostgreSQL database.

**DigitalTwinUtility** - We built a customized Internet of Medical Things (IoMT) utility named DigitalTwinUtility, which runs continuously and interacts with the Patient Data File and the smart contract (DTSmartContractController) to store and retrieve data on the Ganache blockchain and PostgreSQL database. The DigitalTwinUtility (which is the virtual twin of the patient) performs bi-directional communications with the patient and is scheduled to run every 30 days or whenever there is a change in the Patient Data File.

#### Critical-illness ML model

The ML model we used was trained using the Scikit-learn open-source machine learning library, which supports both supervised and unsupervised learning. It also provides various tools for model fitting, data preprocessing, model selection, classification, model testing, and many other utilities. Scikit-learn is widely regarded as the most powerful and popular library for machine learning in Python.

Our approach includes employing the Logistic Regression algorithm, performing Univariate Feature Selection, and optimizing the model using Batch Gradient Descent.

##### Logistic regression algorithm

Logistic regression was chosen for its suitability in binary classification tasks and its flexibility to extend to the prediction of multiple pathologies such as heart attacks, cancers, osteoporosis, or epilepsy.

##### Univariate feature selection

This method is selected for its simplicity, computational efficiency, and effectiveness in reducing overfitting by selecting only the most relevant features.

##### Batch gradient descent optimization process

Batch Gradient Descent updates the model parameters by computing gradients from the entire training dataset. Its simplicity, stability, and ability to handle large datasets make it a suitable choice for optimizing the Logistic Regression model for stroke prediction.

This combination of Logistic Regression, Univariate Feature Selection, and Batch Gradient Descent offers a robust and interpretable approach to predicting pathologies such as brain strokes, aligning well with the requirements of healthcare applications.

#### Hardware and software configurations and versions

In this section, we outline the hardware and software configurations, along with their respective versions, utilized in our experimental set-up for the Digital Twin application.

##### **Server configuration**

- Amazon EC2 Mac instances with x86-based architecture.
- 8 vCPUs and 32 GB RAM allocated to handle the computational demands of heart attack prediction models, including processing real-time data from wearable devices and large-scale patient datasets.
- 10 Gbps networking bandwidth for high-speed communication between server components, external services, and connected heart monitoring devices.

##### **Storage configuration**

- Amazon Elastic Block Store (EBS) with 100 GB capacity for storing patient medical records, real-time heart monitoring data, and predictive model outputs.
- Total storage capacity of 30 GB allocated for data management purposes.
- Daily automated backups to store patient data and models securely, ensuring compliance with healthcare regulations and preventing data loss.

##### **Software versions**

- Ganache: Private Ethereum blockchain environment (6.12.1).
- Solidity: Object-oriented programming language for smart contract development (0.8.11).
- ReactJS: JavaScript library for building user interfaces (17.0.2).
- Scikit-learn: Machine learning library for Python (0.24.1).
- PostgreSQL: Relational database management system (13.3).
- Python: Programming language used for backend development (3.9.5).

### Digital twin application

**Fig. 1:**
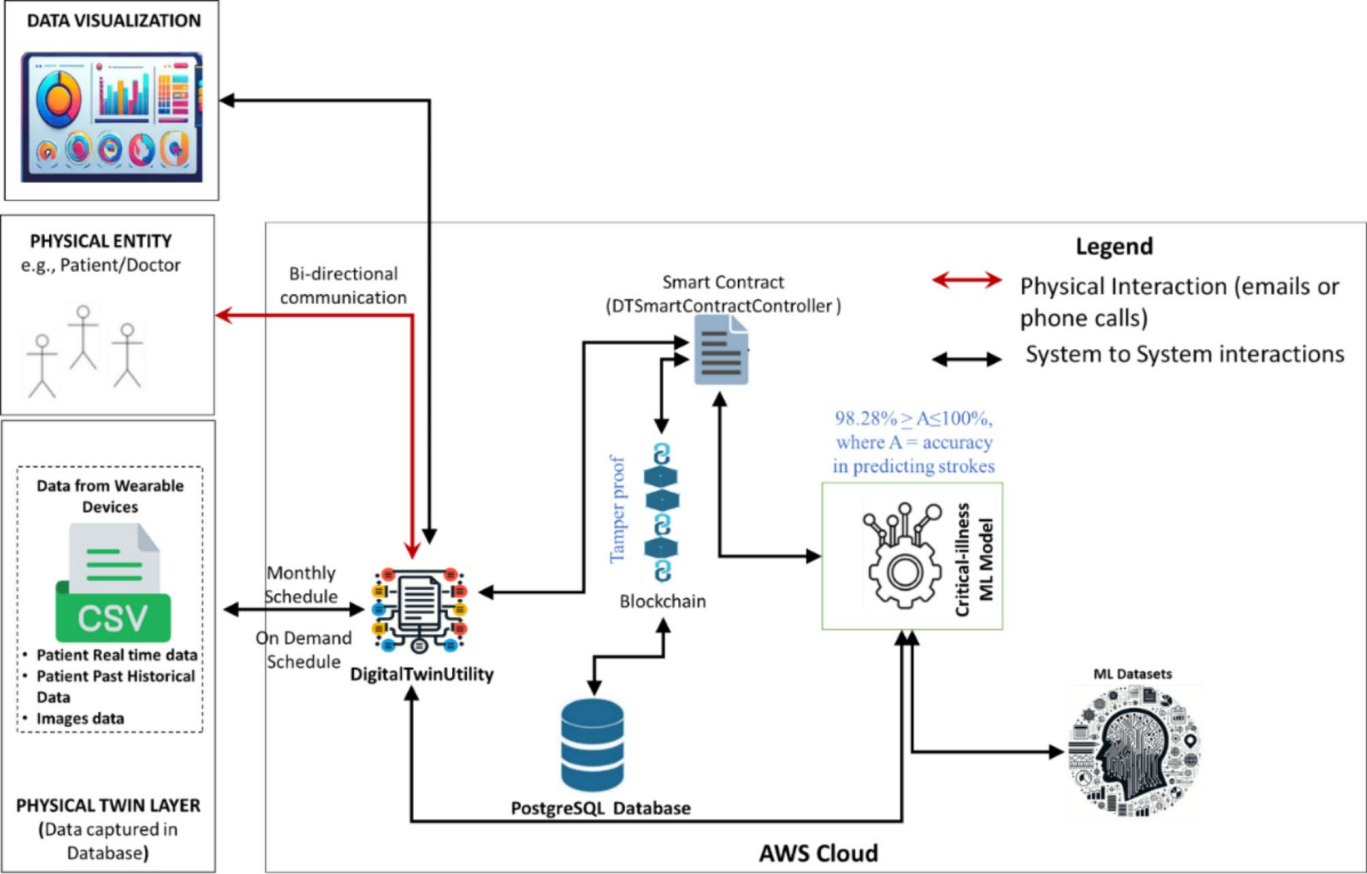
Digital twin prototype (Architecture)

We developed a Digital Twin application model to monitor patients and predict brain strokes. This model utilizes a machine learning algorithm to analyze historical data for assessing the risk of brain strokes. While the focus of this study is on brain stroke prediction, the prototype is designed to be highly adaptable and can be extended to predict other conditions, such as heart attacks, cancers, osteoporosis, and epilepsy. The architecture of the Digital Twin application is depicted in Fig. 1.

The process begins with data retrieval. According to the guidelines from the Joint National Committee on Detection, Evaluation, and Treatment of High Blood Pressure, hypertension levels should be validated approximately every month to reduce the risk of strokes [33]. Following these guidelines, we have developed the application such that patient data is retrieved from the Patient Data File every month, or whenever new readings are available (e.g., blood pressure or cholesterol levels). The Patient Data is stored in XML format to ensure easy integration with future data sources, including wearable devices.

Based on a predefined monthly schedule, the DigitalTwinUtility retrieves patient data from the Patient Data XML File. The data retrieved includes age, sex, past history of heart disease, brain stroke, work type, residence type, smoking status, marital status, Body Mass Index (BMI), hypertension, and average glucose levels. The data is then formatted and sent to the blockchain to ensure it is securely stored. The blockchain plays a crucial role in maintaining the security and accuracy of patient data. It generates a unique identifier for each data entry and stores this along with the patient’s information in a secure database. Once the data is securely stored, the machine learning model analyzes it to predict the risk of a stroke. If the system predicts a high risk of stroke, it automatically notifies healthcare providers via email. Doctors can then use the system’s user-friendly interface to review the patient’s health data and prediction results. The application’s flexible design makes it easy to adapt for predicting other illnesses and integrating new medical devices, making it a valuable tool in modern healthcare.

#### Logistic regression algorithm

The Logistic Regression algorithm has been used as part of this prototype because it is highly adaptable and can be applied to other pathologies including heart attacks, osteoporosis, epilepsy and similar conditions. Logistic Regression is a general-purpose classification algorithm well-suited for binary outcomes, such as predicting whether a specific event (e.g., stroke, heart attack, or epileptic seizure) will occur.

#### Consortium Blockchain for data integrity

We employ a consortium blockchain model with two nodes, simulating a network of two hospitals, to secure patient data and ensure its integrity within our Digital Twin application. This blockchain model enhances security by preventing unauthorized access or tampering, with each node sharing responsibility for data validation. The integration of the consortium blockchain within the architecture is outlined below:

##### Smart contracts

The application utilizes smart contracts, written in Solidity, to automate data integrity checks. Each time new patient data is added or updated, the smart contract generates a unique hash (a cryptographic fingerprint) for that data. This hash is then stored on the blockchain, ensuring data authenticity. Both nodes (representing hospitals) must agree on the validity of the data through a consensus mechanism, preventing tampering before the data is finalized on the blockchain.

##### Data storage

The full patient data, along with its corresponding hash, is stored in a PostgreSQL database. Only the metadata, such as the hash keys and timestamps, is stored on the blockchain. This ensures that while the data integrity is maintained, the blockchain remains lightweight by not storing the entire dataset on-chain. The hybrid approach allows for efficient data management while ensuring the security benefits of the blockchain.

##### Tamper detection

Each time the system retrieves patient data for processing (e.g., predicting stroke risk), the hash stored in the PostgreSQL database is compared with the hash on the blockchain. Since the blockchain is shared between the two nodes, any modification or tampering attempt will be immediately flagged by a mismatch in the hashes. The blockchain’s distributed nature ensures that both nodes must agree on the data’s integrity, and any unauthorized changes will be rejected.

##### Immutable ledger

Due to the blockchain’s immutable nature, once a transaction—comprising patient data and its associated metadata—is recorded, it cannot be altered or deleted. This ensures that a patient’s medical history is preserved in a secure and verifiable manner. Any attempt to tamper with past data would break the cryptographic chain of blocks, alerting both nodes and preventing fraudulent changes.

By employing a consortium blockchain with two nodes, we ensure decentralized control and data validation, making it virtually impossible for a single entity to tamper with patient records. The consensus mechanism between nodes enhances trust and ensures that patient data is recorded accurately and securely. This approach, combined with our smart contract-based validation process, ensures robust data integrity and security across the system.

#### Application scalability

The architecture is adaptable to detecting various pathologies, such as heart attacks, cancers, osteoporosis or epilepsy, by simply adjusting the features used for prediction. This adaptability enables the algorithm to be applied across a broad range of conditions, with each requiring specific patient data to assess the likelihood of occurrence or disease progression. While Logistic regression is highly versatile for many binary classification problems, it has limitations when applied to certain medical pathologies, particularly those with more complex relationships between variables or when the problem is not binary. For example, Parkinson’s Disease, may require a multiclass classification model. In such cases, the application can be reused with minimal modifications, such as updating the machine learning model reference and adjusting the relevant patient data fields to fit the new model requirements.

The Digital Twin application also supports seamless integration with wearable medical devices that provide real-time patient data. Devices that monitor metrics like blood pressure, glucose levels, and other vital signs can automatically write this data to the Patient Data File. Because the system architecture is loosely coupled, new devices can be integrated without altering the core application code, ensuring adaptability and scalability across a wide range of medical technologies.

#### Data visualization and user interface

Two user interfaces (Figs. 4) were developed to provide healthcare providers with a clear view of patient data. These UIs allow doctors to:

- Review patient health metrics and trends over time.
- Receive alerts about patients who are at high risk of stroke.
- Access detailed insights into the ML-predicted risk factors for each patient.

## Results

This section presents the results of our prototype, highlighting its performance in terms of prediction accuracy, scalability, and security.

### Digital twin application accuracy

The likelihood of brain stroke in a patient is detected under the following conditions:

- Hypertension is identified (indicated by a value of 1).
- The presence of heart disease is confirmed (indicated by a value of 1).
- Resting systolic blood pressure exceeds 180 mmHg.
- Serum cholesterol level is greater than 240 mg/dL.
- Fasting blood glucose level falls within the range of > 100 to 125 mg/dL, indicating prediabetes.
- The patient’s maximum heart rate exceeds the predicted maximum based on age. The predicted maximum heart rate is determined by subtracting the patient’s age from 220. For example, a 35-year-old’s predicted maximum heart rate is 185 beats per minute; exceeding this rate during physical activity is considered risky.
- The OL (overshoot level) Peak exceeds 123.
- The SL (segment level) Slope is greater than 2 mm (up), indicating significant ST-segment changes.

These parameters serve as major indicators for assessing the risk of a brain stroke.

We analyzed 62 patient records. Refer to Table 6 in the Appendix for a reference on the structure of individual patient data. Two additional columns on the right side of the table evaluate: the real risk of a patient having a brain stroke and the accuracy of the machine learning (ML) model in predicting the risk of a brain stroke.

To address potential biases in the dataset, we applied fairness evaluation techniques. Fairness evaluation techniques refer to methods used to assess the fairness of AI systems, particularly in terms of their impact on different demographic groups, such as race, gender, age, or socioeconomic status. These techniques aim to identify and mitigate biases and discriminatory effects that AI systems may exhibit.

We performed two additional experiments involving different datasets categorized by patient age: one with patients aged over 70, another with patients aged between 40 and 70, and a third with patients under 40. The outcomes are presented in Table 5, revealing that the machine learning model effectively identified individuals at risk of brain stroke with a prediction accuracy exceeding 98.28%.

**Table 5:** Prediction results across multiple experiments

Dataset Type	Reference to the structure of individual patient data	Prediction Accuracy %
Masked Patient Data set aged > 70	Appendix A - Table 7	100%
Masked Patient Data set aged < 70 and > 40	Appendix A - Table 8	98.28%

Based on the aggregation of all available results, the Human Digital Twin (DT) model we developed has demonstrated a prediction accuracy of 98.28% in identifying the risk of brain strokes.

### Digital twin application scalability

The Digital Twin application uses a scalable Logistic Regression model to predict the risk of brain stroke and can be adapted to other critical illnesses, such as heart attacks (by using features like cholesterol levels, blood pressure, and family history) and epilepsy (using features such as BMI, blood sugar levels, and family history) allowing it to be effectively used for various conditions with no code changes to the Digital twin application.

For pathologies that cannot use binary classification logistic regression model, there are just few steps, as described below, to extending our digital twin application to work for critical illness that requires complex computations.

**Add new fields (If required)** –If a new illness requires additional patient fields in the database, update the PatientData.xml file with the necessary fields.

**Modify the ML model reference** – To configure the application for predicting a different critical illness, update the model reference in the configuration.xml file to point to the appropriate ML model for the new illness.

**Restart the application** – After making the necessary configuration changes, restart the application to activate the prediction capabilities for the new illness.

This process highlights the Digital Twin application’s scalability in adopting to any other use cases.

#### Integration with other medical devices

As depicted in Fig. 1, we have used CSV files to capture patient real-time data such as resting average blood pressure, average cholesterol level, average fasting blood sugar levels, average resting electrocardiography, average maximum heart rate, exercise-induced angina, and hypertension levels. The application we have developed is loosely coupled from the patient data file (CSV files), as shown in Fig. 1 above. This setup enables seamless integration with other medical devices. The wearable devices simply need to be configured to write the data directly into the CSV files. No other changes are required to the application, and the model we have developed will work seamlessly regardless of the diversity of medical devices that need to be integrated with the application. This feature highlights the adaptability and scalability of our application to integrate with medical devices.

### Simulating and detecting data tampering in a consortium blockchain

The security of the system and the health and safety of patients could be compromised in the event of a cyberattack. To test the resilience of our consortium blockchain setup, we simulated a data tampering attack by a malicious user targeting patient data stored in both the Ganache consortium blockchain and the PostgreSQL database. In this simulation, we deliberately altered patient data to invalid values, mimicking a typical cyberattack.

The original data for Patient 6 included:

Body Mass Index (BMI) = 18.

Hypertension Level = 100.

Average Glucose Level = 100.

These values indicated no immediate risk of brain stroke. In the tampering scenario, we changed the data from back end to:

Body Mass Index (BMI) = 146.

Hypertension Level = 120.

Average Glucose Level = 150.

These altered values now implied that Patient 6 was at a higher risk for brain stroke.

#### Tampering detection via consortium blockchain

In our consortium blockchain model, where two nodes (representing hospital organizations) share control of the blockchain, such data tampering attempts are quickly identified. The blockchain operates through a consensus mechanism, meaning both nodes must validate the integrity of the data before it is accepted into the ledger. In this case, the tampered data caused a mismatch in the cryptographic hash, as the original values stored on the blockchain did not match the altered data in the PostgreSQL database.

Upon detecting this discrepancy, the blockchain system immediately flagged the tampered transaction. The smart contract automatically rejected the invalid data and reverted the records back to their original state, as validated by the hashes stored across both nodes of the consortium blockchain.

**Fig. 2:**
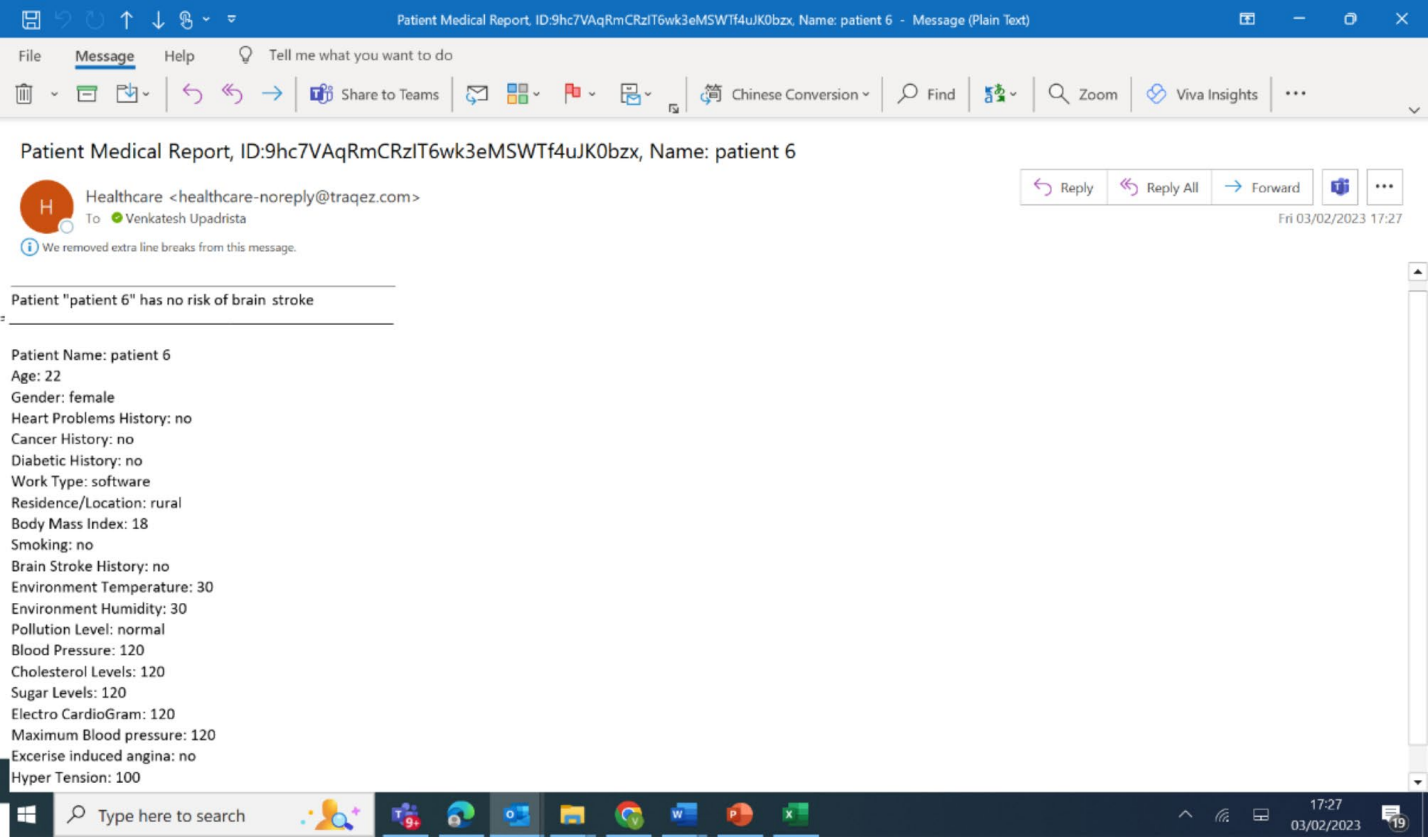
Email Notification retaining the original values even though patient data was tampered from backend

Additionally, as part of our alert system, an email notification (refer Fig. 2) was automatically triggered, informing the relevant administrators of the data tampering attempt. The email highlighted the discrepancy and reassured that the original values were retained across the blockchain network, preventing any unauthorized changes from being committed. This real-time alert system provides transparency and ensures that the stakeholders are promptly notified of any malicious activity while maintaining data integrity.

### Data visualization

**Fig. 3:**
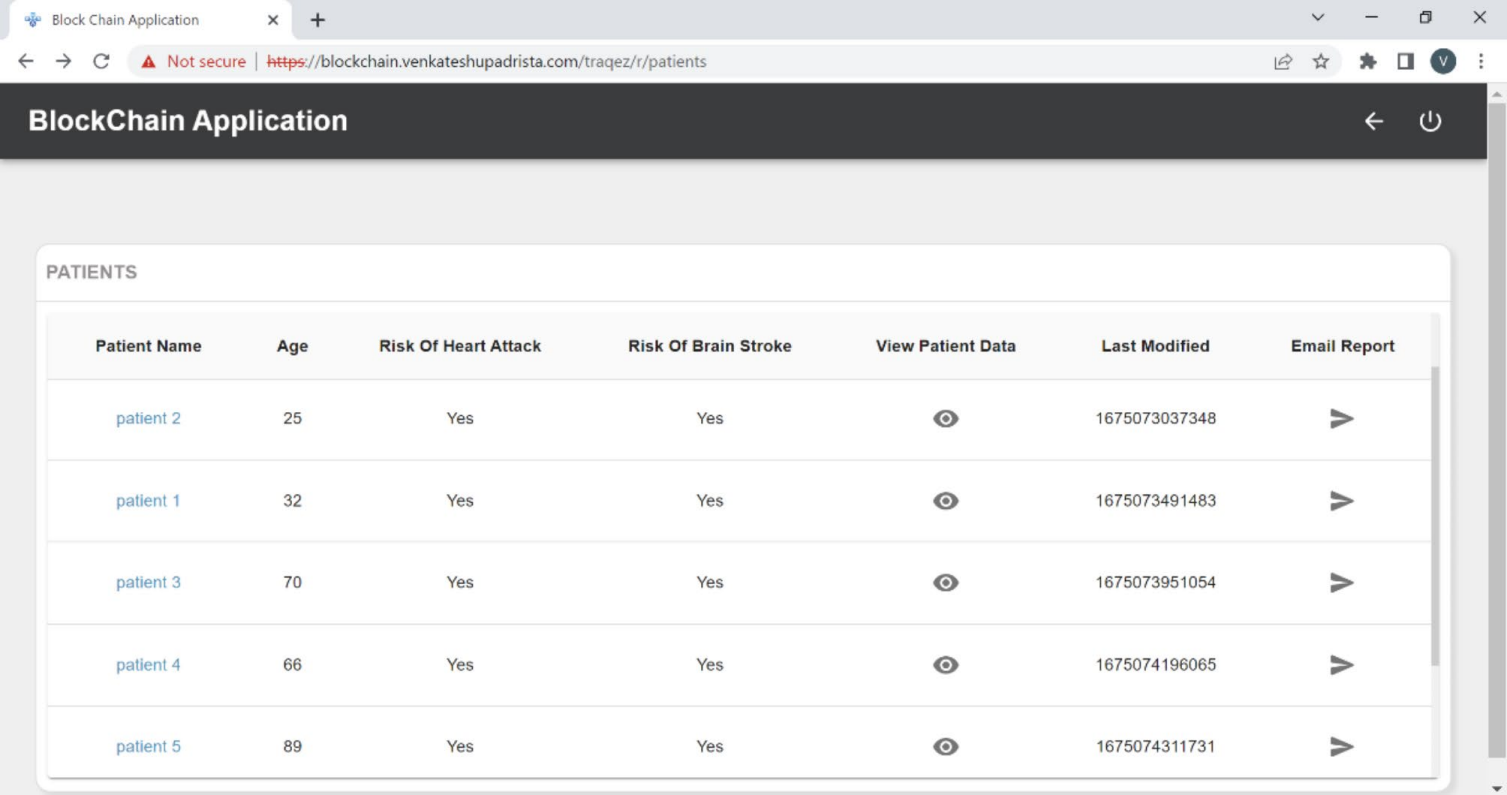
UI 1 - Patients summary user interface

**Fig. 4:**
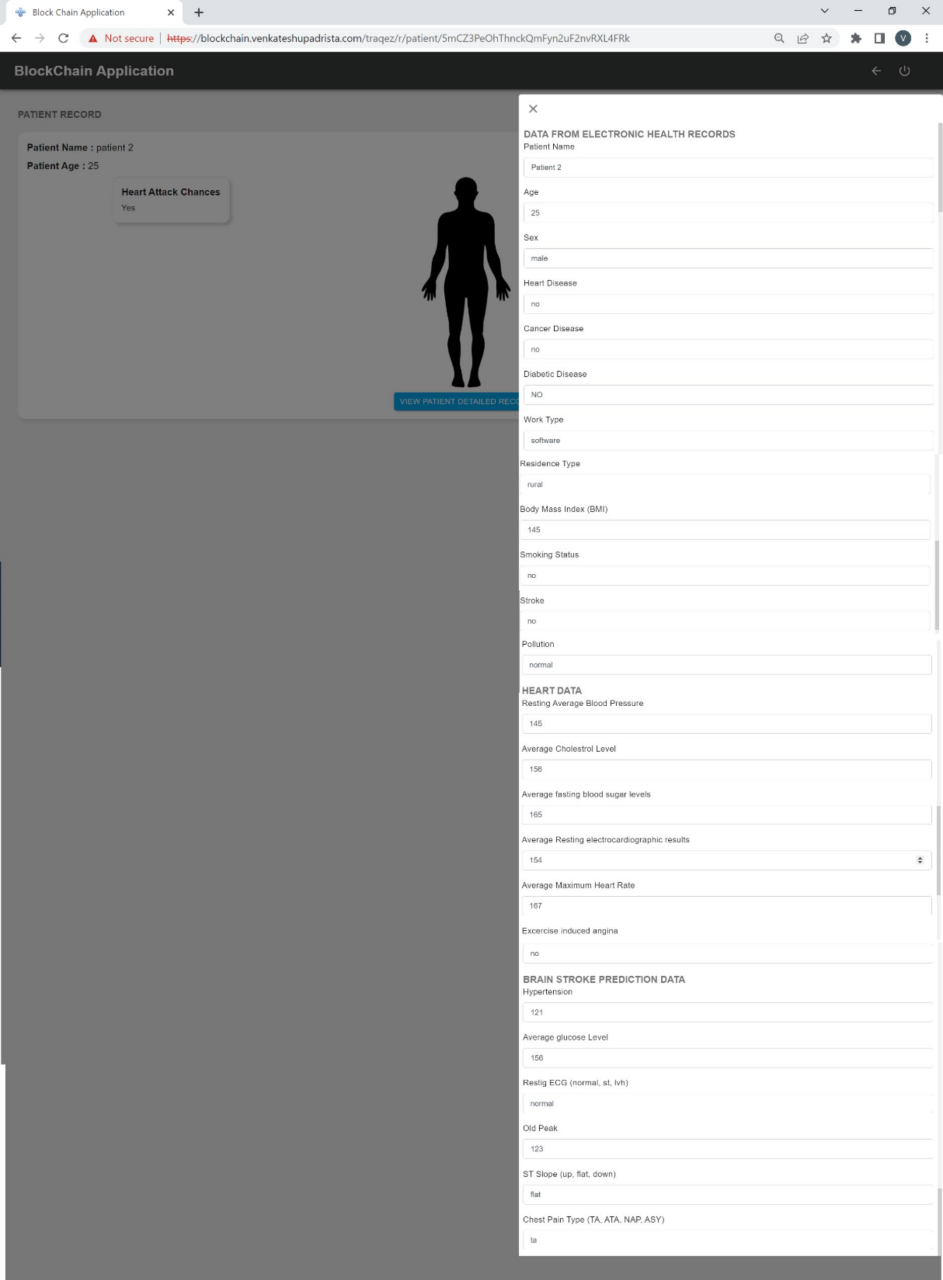
UI 2 - Individual patient data

Within our Digital Twin application, doctors will primarily receive email notifications about a patient’s brain stroke risk. However, in some cases, they may want to review detailed real-time health data, including the patient’s medical history from electronic health records. To address this, we have developed two alternative user interface flows (Figs. 3 and 4) that allow doctors to access comprehensive medical reports for each patient.

#### Patient summary report

The UI displaying all patient summary data is presented in Fig. 3, where the doctor can access records of all their patients, including any associated brain stroke risks.

#### Detailed patient data

The doctor can access the details of each patient data by clicking on the individual patient’s name.

### Summary of results

We have demonstrated the effectiveness of our prototype in terms of prediction accuracy, scalability, and security. Our application achieved a prediction accuracy of 98.28% in identifying the risk of brain strokes. This performance exceeds the accuracy levels reported in the literature, which typically range from 84 to 92% [9–15, 22–25]. However, further rigorous validation is necessary to evaluate our model’s performance against diverse, real-world datasets, including those used in these literature studies.

Additionally, our model showcased superior scalability, as it can be easily extended to predict other critical illnesses like heart attacks, cancers, osteoporosis, and epilepsy without requiring modifications to the core application code, which was not spoken about in the literature so far.

In terms of security, the integration of consortium blockchain technology ensures robust protection of sensitive patient data. This approach goes beyond the methods commonly employed in the literature, such as basic encryption techniques [10, 15, 23] access control mechanisms [14, 22, 25] and data anonymization practices [12, 27, 29] providing enhanced resilience against cyberattacks.

## Discussion

Security and privacy concerns are significant challenges in healthcare [34–38], and many digital twin implementations in the literature have not adequately addressed these issues. Additionally, the models discussed in the literature for detecting pathologies such as heart attacks and brain strokes are often designed specifically for a single condition, limiting their ability to detect other pathologies and restricting their scalability. Moreover, the observed accuracy levels in the studies reviewed so far have yet to surpass the 92% accuracy threshold [9–11, 22–24].

In this paper, we present a Digital Twin application designed to be easily extendable for various critical illnesses with minimal changes to the existing code. Initially tested for brain stroke prediction using the logistic regression algorithm, the application can be seamlessly adapted for other conditions such as heart attacks, cancers, osteoporosis, or epilepsy without further modifications. Additionally, it can handle more complex diseases where binary classification is insufficient, with detailed steps provided to ensure full scalability. The application can also be effortlessly expanded to integrate wearable devices, enabling the transmission of real-time patient data. The model has demonstrated an accuracy of 98.28% under controlled conditions. To address security and privacy concerns, the model incorporates a Consortium blockchain-based solution, making it highly secure. We tested the application by deliberately tampering with backend data, and the system successfully detected the tampering and restored the original values. Smart contracts, written in Solidity, were employed to manage data validation and integrity checks across the nodes. These smart contracts automatically triggered corrective actions when discrepancies were found between the tampered data and the original metadata stored on the blockchain. Instead of storing all patient data on the blockchain, only patient metadata is stored, resulting in a lightweight and efficient blockchain implementation, unlike other approaches in the literature where all data is stored on the blockchain.

While the model has demonstrated an accuracy of 98.28%, a limitation of this study is the use of a small and curated dataset for brain stroke prediction and illustration of system’s scalability to other healthcare use cases. Although the study highlights the accuracy, security, and scalability of the proposed system, additional experiments and comparative analyses are necessary to substantiate these advantages fully. This includes evaluating the system against datasets referenced in existing literature to determine if it can consistently surpass the accuracy levels reported in prior studies.

In summary, the model presented in this paper can be used to address three key aspects: high predictive accuracy, robust security, and scalability for other pathologies with minimal modifications to the application code.

## Conclusion and future work

This paper introduces a Digital Twin application integrated with a machine learning model for predicting pathologies such as heart attacks, cancers, osteoporosis and epilepsy. Unlike other models that focus on specific organs or single-use cases, our application replicates the entire human body, making it adaptable for predicting a wide range of medical conditions. Through minor configuration adjustments, the model can seamlessly predict other diseases without the need for code modifications. Security and privacy are addressed comprehensively in our model through the integration of consortium blockchain technology. This ensures robust protection of sensitive healthcare data, ensuring data. Additionally, our machine learning model achieved a prediction accuracy of 98.28%. Our application also features a loosely coupled architecture, allowing seamless integration with various medical devices and ensuring compatibility with existing healthcare infrastructures.

Future work will validate the application performance using real-world datasets to ensure its robustness and reliability in practical applications. We also plan to extend the Digital Twin model by integrating it with various wearable devices, such as blood pressure and heart rate monitors. Additionally, we aim to enhance the application’s security by implementing Advanced Encryption Standard (AES) across both the device layer and the cloud platform. Given the blockchain’s decentralized transactions higher latency than traditional databases, we will consider employing off-chain solutions for faster transactions, in conjunction with sharding techniques to enable parallel processing across smaller blockchain segments.

## Data Availability

The datasets generated during and/or analyzed during the current study are retrieved from Kaggle https://www.kaggle.com/datasets/jillanisofttech/brain-Stroke-dataset. Kaggle is one of the most reliable and authentic platforms for data scientists and researchers, recognized by reputable sources such as Forbes [39]. We have created real time patient data which constitute of patient individual readings (e.g., blood pressure, Heart rate, brain related readings) which can be downloaded from https://github.com/vupadrista/Blockchain-Enabled-Digital-Twin-System-for-Brain-Stroke-Prediction. All appendices referenced in this paper are available at https://figshare.com/articles/journal_contribution/Blockchain-Enabled_Digital_Twin_System_for_Brain_Stroke_Prediction/27980363?file=51027824

## Code availability

The code used during and/or analysed during the current study is available at https://github.com/vupadrista/Blockchain-Enabled-Digital-Twin-System-for-Brain-Stroke-Prediction.

## Abbreviations

API: Application Programming Interface
IoMT: Internet of Medical Things
RPM: Remote Patient Monitoring
XML: Extensible Markup Language
EHR: Electronic Health Record
ML: Machine Learning
ST Slope: ST Segment Slope (used in cardiac monitoring)
ECG: Electrocardiography
BMI: Body Mass Index
GAN: Generative Adversarial Network (not directly mentioned but often abbreviated in related contexts)
AES: Advanced Encryption Standard
OL Peak: Overshoot Level Peak
SL Slope: Segment Level Slope
SPoF: Single Point of Failure
Ref.: Reference number of the study
Acc.: Accuracy level of the model
Sec. Measures: Security measures implemented
Encrypt.: Encryption
Access Ctrl.: Access Control
Data Anonym.: Data Anonymization
Hom. Encryption: Homomorphic Encryption
Proto. Sec.: Prototype for Security/Privacy
Temp. Conv. Networks: Temporal Convolutional Networks
Geo., Temp.: Geographical, Temporal
Feature Sel.: Feature Selection
